# Ca^2+^/Calmodulin-Dependent Protein Kinase Kinases (CaMKKs) Effects on AMP-Activated Protein Kinase (AMPK) Regulation of Chicken Sperm Functions

**DOI:** 10.1371/journal.pone.0147559

**Published:** 2016-01-25

**Authors:** Thi Mong Diep Nguyen, Yves Combarnous, Christophe Praud, Anne Duittoz, Elisabeth Blesbois

**Affiliations:** 1 INRA, UMR85 Physiologie de la Reproduction et des Comportements, F-37380 Nouzilly, France; 2 CNRS, UMR7247, F-37380 Nouzilly, France; 3 Université François Rabelais de Tours, F-37000 Tours, France; 4 IFCE, F-37380 Nouzilly, France; 5 INRA, Unité de Recherches Avicoles, F-37380 Nouzilly, France; Cornell University College of Veterinary Medicine, UNITED STATES

## Abstract

Sperm require high levels of energy to ensure motility and acrosome reaction (AR) accomplishment. The AMP-activated protein kinase (AMPK) has been demonstrated to be strongly involved in the control of these properties. We address here the question of the potential role of calcium mobilization on AMPK activation and function in chicken sperm through the Ca^2+^/calmodulin-dependent protein kinase kinases (CaMKKs) mediated pathway. The presence of CaMKKs and their substrates CaMKI and CaMKIV was evaluated by western-blotting and indirect immunofluorescence. Sperm were incubated in presence or absence of extracellular Ca^2+^, or of CaMKKs inhibitor (STO-609). Phosphorylations of AMPK, CaMKI, and CaMKIV, as well as sperm functions were evaluated. We demonstrate the presence of both CaMKKs (α and β), CaMKI and CaMKIV in chicken sperm. CaMKKα and CaMKI were localized in the acrosome, the midpiece, and at much lower fluorescence in the flagellum, whereas CaMKKβ was mostly localized in the flagellum and much less in the midpiece and the acrosome. CaMKIV was only present in the flagellum. The presence of extracellular calcium induced an increase in kinases phosphorylation and sperm activity. STO-609 reduced AMPK phosphorylation in the presence of extracellular Ca^2+^ but not in its absence. STO-609 did not affect CaMKIV phosphorylation but decreased CaMKI phosphorylation and this inhibition was quicker in the presence of extracellular Ca^2+^ than in its absence. STO-609 efficiently inhibited sperm motility and AR, both in the presence and absence of extracellular Ca^2+^. Our results show for the first time the presence of CaMKKs (α and β) and one of its substrate, CaMKI in different subcellular compartments in germ cells, as well as the changes in the AMPK regulation pathway, sperm motility and AR related to Ca^2+^ entry in sperm through the Ca^2+^/CaM/CaMKKs/CaMKI pathway. The Ca^2+^/CaMKKs/AMPK pathway is activated only under conditions of extracellular Ca^2+^ entry in the cells.

## Introduction

Biological sperm functions such as motility and ability to undergo acrosome reaction (AR) are central to male fertility. These functions are highly dependent on energetic metabolism which is itself largely controlled by 5’-AMP activated protein kinase (AMPK) signaling. The activity of this kinase is regulated by calcium through signaling pathways [1–2] that are not yet determined in chicken sperm. Bird fertilization exhibits numerous specificities comprising oviparity, complex internal fertilization and long term sperm storage in specific oviductal storage tubules, making it a unique model in fertilization studies [3–4] and important for the sake of comparison with other vertebrate species. Chicken sperm also shows very rapid signaling reactions that make them unique for metabolic signal transduction studies and was chosen as model for the present studies [5–6].

The PKA, PKB and PKC signaling pathways have previously been shown to be involved in chicken motility regulation [7–8] and PKA, PI3K and ERK2 have proved to be key proteins for chicken sperm AR [5]. We have recently demonstrated the involvement of AMPK in chicken sperm regulation of motility and acrosome reaction [6]. Nevertheless, relationships between sperm functions regulation by AMPK and calcium signaling remained to be explored.

The Ca^2+^/calmodulin-dependent protein kinase kinases (CaMKKs) were initially identified as novel members of the protein serine/threonine kinases CaMK family, with 2 forms, CaMKKα and CaMKKβ (also named CaMKK1 or CaMKK2, respectively), both expressed in the nervous system, in endothelial cells of many areas of the brain, in hematopoietic cells, and at lower levels in testis, spleen, lung, liver and skeletal muscle [9–14]. In other tissues, such as kidney, intestine, and heart, the evidence for expression remains less clear [11–12, 14]. Upon interaction with calcium-bound calmodulin (Ca^2+^-CaM), CaMKKs activate two calmodulin-dependent protein kinases: CaMKI through phosphorylation at Thr177 and CaMKIV through phosphorylation at Thr196 [15–17]. CaMKKs can also phosphorylate and activate PKB/Akt [18] and AMPK [1–2]. CaMKKβ was identified as being an AMPK kinase which phosphorylates AMPK at Thr172 in response to an increase in intracellular Ca^2+^ [1–2]. CaMKK (α and β) inhibition causes a drop in AMPK phosphorylation in boar sperm [19]. However, the full characterization of the mechanisms involved in the regulation of AMPK phosphorylation and activity in sperm, including those involving CaMKKs, remains to be explored.

Calcium signaling pathways are essential in regulating cellular processes such as muscle contraction, neurotransmitter release, cellular metabolism, gene expression, and cell proliferation [20–21]. In sperm, Ca^2+^ plays a prominent role during fertilization in all animal species. In mammals, extracellular Ca^2+^ is required for epididymal acquisition of sperm motility in mice, rats, pigs, hamsters and bovines [22–26], and is known to regulate both activated and hyperactivated motility [27–29]. Calcium controls flagellar motility through the regulation of dynein-driven microtubule sliding and modulation of the sperm tail waveform [30–31]. It also plays a central role in the acrosome reaction in invertebrates such as echinoderms and in superior vertebrates such as birds [32] and mammals [33–34].

The Ca^2+^/CaM complex regulates the activity of multiple enzymes, including adenylate cyclases [35], phosphatases [36], and protein kinases [19]. The CaMKs are among the signaling proteins regulated by Ca^2+^/CaM, and are present in most mammalian tissues: thymus, bone marrow, ovary and testis [37], and are abundant in brain. CaMKII has been immunolocalized in the acrosomal region and the flagellum of demembranated bovine sperm and has been shown to stimulate hyperactivation [38] and, more widely, to regulate motility [39]. CaMKIV has been localized in the intermediate piece and the flagellum of human sperm [40]. CaM plays a role in mouse sperm capacitation [41]. In fowl sperm, it has been suggested that the increase in intracellular free Ca^2+^ and CaM is necessary for the maintenance of sperm motility [42] and Lemoine *et al*. (2009) have shown that extracellular Ca^2+^ is essential for the initiation of chicken acrosome reaction [32]. However, the Ca^2+^/CaMKKs/AMPK pathway and their effects on sperm functions remain to be clearly identified.

The aim of this study was to examine the mechanisms involved in the Ca^2+^/ CaMKKs/CaMKI signaling pathway upstream of AMPK activation in chicken sperm. We studied the immunolocalization of CaMKKs (α and β) and their downstream kinase CaMKI, the effects of CaMKK inhibitor (STO-609) and extracellular Ca^2+^ on AMPK and CaMKI phosphorylation. We also suggested the involvement of Ca^2+^/CaMKKs/AMPK and Ca^2+^/CaM/CaMKKs/CaMKI signaling pathways in the regulation of chicken sperm functions.

## Materials and Methods

### Chemicals and reagents

All chemicals were purchased from Sigma–Aldrich (St Louis, MO, USA) unless otherwise noted. Complete mini EDTA-free, protease inhibitor cocktail tablets were from Roche diagnostics (Mannheim, Germany). Tris/glycine buffer (10X), Tris/glycine/SDS buffer (10X), and Precision Plus Protein All Blue Standards (Catalog #161–0373) were obtained from Bio-Rad (Hercules, CA) and anti-AMPKα from Millipore (Billerica, MA), anti-phospho-Thr172-AMPKα, anti-CaMKIV, and anti-rabbit IgG (H+L) (DyLight 680 Conjugate) from Cell Signaling Technology, Inc (Danvers, MA). Anti- CaMKKβ and anti-CaMKKα were obtained from Abgent INC (San Diego, CA). Anti-CaMKI and anti-phospho-Thr177-CaMKI were obtained from Novus Europe (Cambridge, UK). Anti-phospho-Thr196-CaMKIV was obtained from Santa Cruz Biotechnology, INC (Texas, USA). PI/Sybr-14 (LIVE/DEAD sperm viability kit) was purchased from Molecular Probes (Saint Aubin, France).

### Animals and semen collection

All experiments were carried out in accordance with the legislation governing the ethical treatment of animals and approved by the Ethics Committee (“Comité d'Ethique en Expérimentation Animale du Val de Loire”, Tours, France, N° 19).

The animals were 22 to 55 weeks-old adult chickens of the D+/D- line [43]. All the animals were housed in individual battery cages under a 14L/10D photoperiod and fed with a standard diet of 12.5 MJ/day.

The semen was collected by dorso-abdominal massage [44]. This technique does not harm the animals: they are simply caught by hand and free to go after the abdominal massage, without suffering any injury. The semen from the four males were then mixed together and centrifuged for 10 min at 600g at 20°C [5, 32]. Sperm pellets were resuspended in Lake 7.1 [45] (composition: 5.6mM magnesium acetate, 4.2mM tripotassium citrate, 89.9mM sodium glutamate, 44.4mM glucose, 143mM BES (N, N-bis[2-hydroxyethyl]-2-aminoethanesulfonic acid), 4% NaOH, adjusted to 1L with distilled water; pH 7.1 and osmolality 400-410mOsmol/kg). Concentration of sperm was estimated at a wavelength of 530nm. Concentrations were close to 500 x 10^6^ cells/ml.

### Sperm incubation with modulators of AMPK

Sperm samples were incubated in Lake 7.1 buffer throughout the present work. The Lake 7.1 medium not supplemented with 5mM CaCl_2_ was called “-5mM Ca^2+^”, the Lake 7.1 medium containing maximum amounts of CaCl_2_ was called “+5mM Ca^2+^”. All these media had similar osmolalities (400–410 mOsm) and pH (7.1).

Sperm samples were incubated at 35°C in the presence or absence of 5mM Ca^2+^ with or without different doses of STO-609 (1, 10 and 50μM). Sperm quality (viability, motility parameters, and acrosome reaction) was measured at different times (5, 15, and 30 min). When necessary, a control with the final concentration of the solvent (0.1% DMSO) was included.

### Sperm viability assessment

SYBR-14/PI was used to assess sperm viability [46]. Sperm were diluted in Lake 7.1 buffer down to 20 x 10^6^ cells/ml, 5μl Sybr-14 was added and then the solution was incubated for 10 min in darkness at 4°C. Afterwards, 2μl of propidium iodide (PI) were added and the incubation was continued for 5 min in the dark at 4°C. After incubation, sperm cell viability was assessed by fluorescence microscopy (Zeiss Axioplan 2; Zeiss Gruppe, Jena, Germany): living cells appeared green and dead ones red. A total of 300 sperm per slide were counted (two slides/sample = 1 replicate) and a total of six replicates/treatment were examined. All preparations were analyzed by the same observer.

### Analysis of sperm motility by computer-assisted sperm analysis (CASA) system

The sperm motility parameters were evaluated by the computer-assisted sperm analysis (CASA) system with a HTM-IVOS (Hamilton Thorne Biosciences, Beverly, MA) [47]. In this experiment, the parameters measured were: percentage of motile sperm was defined as the percentage of spermatozoa showing a VAP > 5μm/s, progressive cells were defined as sperm having VAP > 50μm/s and STR > 75%, rapid cells were defined as having VAP > 50μm/s, slow cells were defined as having VAP < 50μm/s, and static cells (sperm not moving at all) were defined as having VAP ≤ 5μm/s.

### Acrosome reaction (AR) assessment with FITC-PNA

The completion of the acrosome reaction was detected by FITC-conjugated peanut agglutinin (FITC-PNA) binding [48]. Sperm (500 x 10^6^ cells/ml) were incubated at 41°C with 50μl of the hen inner perivitelline layer (IPVL) and 500μl of NaCl-TES (TES: N-Tris-[hydroxymethyl]-methyl-2-aminoethanesulfonic acid) (318 mOsm and pH 7.4) containing 5mM Ca^2+^ for 5 min. The samples were then centrifuged at 400g for 5 min and the pellets resuspended in 100μl NaCl-TES. FITC-PNA was then added (1mg/ml) and the sperm were incubated for 10 min in the dark at 4°C, then washed in 440μl of NaCl-TES and centrifuged at 400g for 5 min. The pellets were resuspended in 200μl NaCl-TES for analysis. The sperm having completed their acrosome reaction were identified and counted under fluorescence microscopy (Zeiss Axioplan 2; Zeiss Gruppe, Jena, Germany). A minimum of 100 sperm were counted for each sample (two slides/sample = 1 replicate) and a total of six replicates/treatment examined. Acrosome reaction was characterized by the green fluorescence of the acrosomal region [5, 32]. All preparations were analyzed by the same observer.

### Western–Blotting

For western-blotting experiments, total proteins were extracted from chicken sperm in lysis buffer (10mM Tris, 150mM NaCl, 1mM EGTA, 1mM EDTA, 100mM sodium fluoride, 4mM sodium pyrophosphate, 2mM sodium orthovanadate, 1% Triton X-100, 0.5% NP40 containing a protease inhibitor cocktail with EDTA). Cell lysates were centrifuged at 12000g for 30 min at 4°C and the protein concentration in each supernatant was determined by a colorimetric assay (Bio-Rad DC Protein Assay; Bio-Rad, Hercules, CA). The proteins were then separated by 10% SDS-PAGE (SDS Polyacrylamide Gel Electrophoresis) and transferred onto nitrocellulose membrane (Whatman Protran, Dassel, Germany). Afterwards, the membranes were incubated in anti-CaMKKα (50kDa), anti-CaMKKβ (65kDa) or anti-AMPKα (62kDa), anti-phospho-Thr172-AMPKα (62kDa) or anti-CaMKI (41kDa), anti-phospho-Thr177-CaMKI (41kDa) or anti-CaMKIV (60kDa), anti-phospho-Thr196-CaMKIV (60kDa) diluted in 5% BSA in TBS-Tween 0.1% (final dilution 1:1000) as primary antibodies overnight at 4°C. Finally, the membranes were further incubated for one hour in anti-rabbit IgG (H+L) (DyLight 680 Conjugate) (final dilution 1:2000). The band intensity was analyzed using Odyssey Software, version 1.2 (LICOR Biosciences, Lincoln, Nebraska, USA). AMPKα, CaMKI, or CaMKIV were used as loading controls. The secondary antibody alone control was used to check for non-specific labeling by the secondary antibody.

### Immunocytochemistry

CaMKKα, CaMKKβ, CaMKI and CaMKIV were localized in chicken sperm by immunocytochemistry. Sperm (500 x 10^6^/ml) were fixed in paraformaldehyde (4%) for 4 min, spread onto a poly-L-lysine slide and then air-dried at room temperature. Afterwards, sperm were washed in PBS (3 x 3 min) and then permeabilized for 10 min with 0.35% Triton X-100 (Sigma-Aldrich) in PBS. Non-specific binding was blocked with PBS supplemented with 10% goat serum (Sigma–Aldrich) for 30 min at room temperature. Samples were then incubated overnight at 4°C with anti-CaMKKα, anti-CaMKKβ, anti-CaMKI or anti-CaMKIV, diluted 1:100 in PBS-1% goat serum, rinsed (3 x 3 min) with PBS and then incubated with biotinylated goat anti-rabbit IgG (H+L) (Southern biotech, USA) (1:200 in PBS-1% goat serum) for 1 hour at 4°C. After 3 rinses with PBS, sperm cells were incubated with Cy2 conjugated-Streptavidin (Southern biotech) (1:200 in PBS) for 45 min at room temperature in the dark, rinsed (3 x 3 min) with PBS and incubated with 4’,6’-diamidino-2-phenylindole (DAPI) (Sigma–Aldrich) (0.05μg/ml) for 10 min. The presence of CaMKKα, CaMKKβ, CaMKI and CaMKIV in sperm was examined by fluorescence microscopy (Zeiss Axioplan 2, Zeiss Gruppe, Jena, Germany). Negative controls were performed by omitting primary antibodies.

### Statistical analyses

The data are expressed as mean ± standard error of the mean (SEM). Statistical analyses were performed using the GraphPad InStat program (GraphPad Software, San Diego, CA). The significance of the difference between treatments was calculated by Student's t-test or analysis of variance (ANOVA). The level of significance was at P < 0.05.

## Results

### Identification and localization of CaMKKs and its substrates CaMKI and CaMKIV in chicken sperm

**Fig 1:**
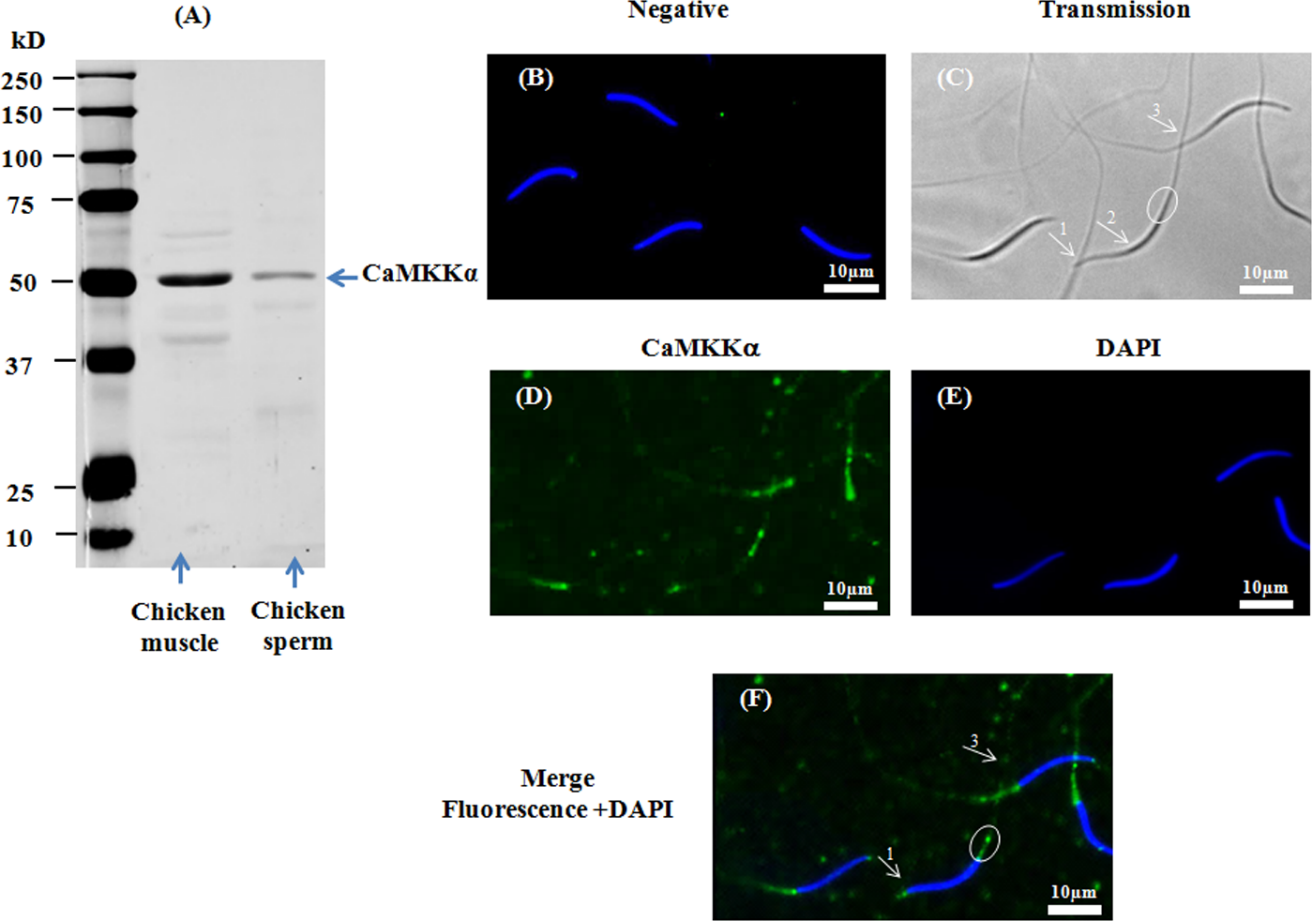
Presence and localization of CaMKKα in chicken sperm. Chicken sperm lysates (45μg of protein) were analyzed by western blotting using anti- CaMKKα as primary antibody. Cell lysates from chicken muscle (40μg of protein) were used as positive control. A band of approximately 50kDa for CaMKKα was detected (**1A**). Indirect immunofluorescence of chicken sperm was carried out with the same antibody. Negative control: primary antibody was not added (**1B**). White arrows and circles in transmission images indicate areas of the acrosome (arrow 1), the nuclei (arrow 2), the midpiece (circle), and the principal piece of the flagellum (arrow 3) in sperm (**1C**). Immunofluorescence staining of CaMKKα (**1D**, green) was conducted; nuclei were stained with DAPI (**1E**, blue). Merged images of fluorescence with DAPI staining are shown in Fig **1F** (white arrows and circles indicate regions containing CaMKKα immunoreactivity: arrow 1: acrosome; arrow 3: principle piece; circle: midpiece). Scale bar: 10μm.

**Fig 2:**
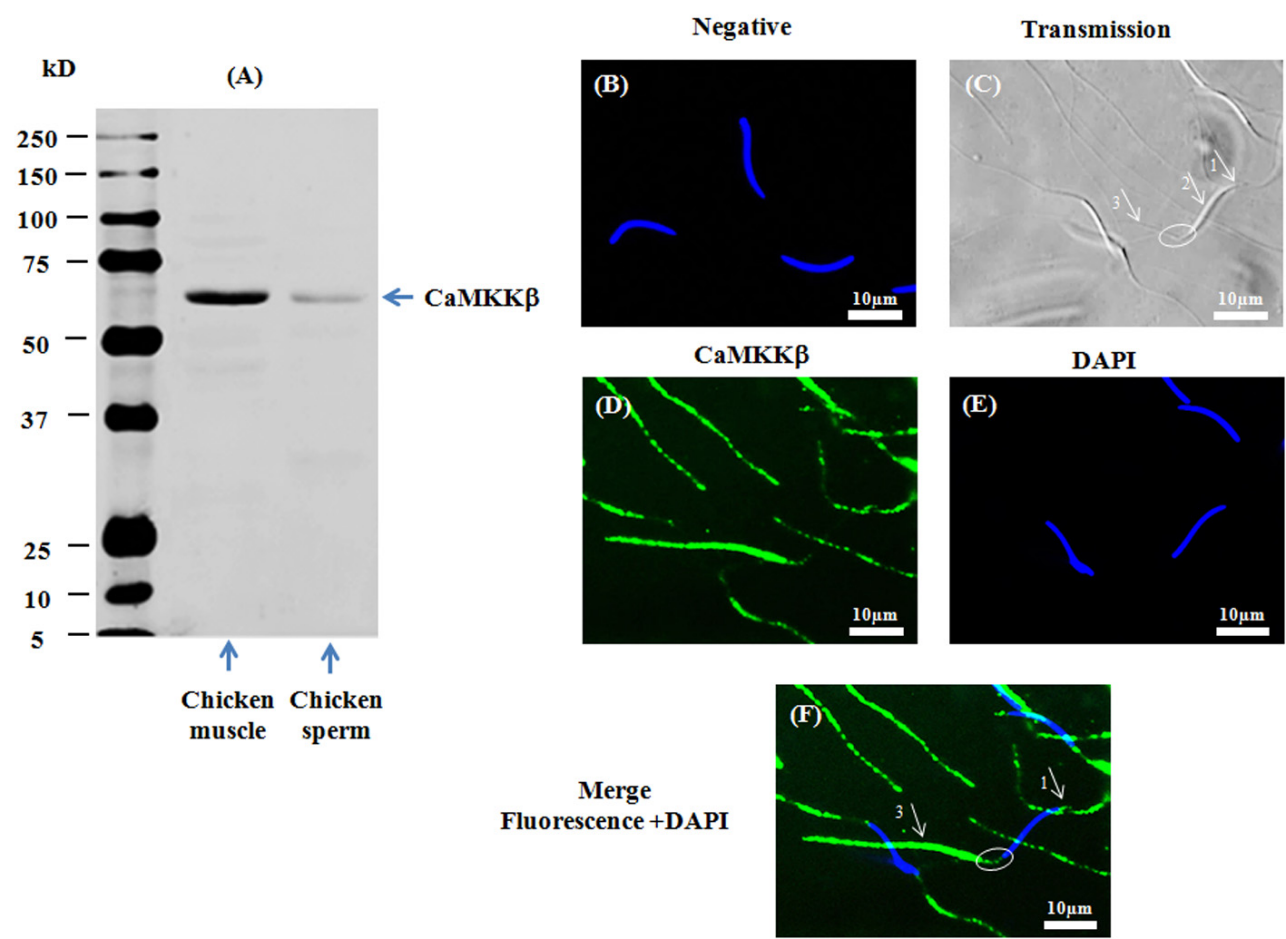
Presence and localization of CaMKKβ in chicken sperm. Chicken sperm lysates (45μg of protein) were analyzed by western blotting using anti- CaMKKβ as primary antibody. Cell lysates from chicken muscle (40μg of protein) were used as positive control. A band of approximately 65kDa for CaMKKβ was detected (**2A**). Indirect immunofluorescence of chicken sperm was carried out with the same antibody. Negative control: primary antibody was not added (**2B**). White arrows and circles in transmission images indicate areas of the acrosome (arrow 1), the nuclei (arrow 2), the midpiece (circle), and the principal piece of the flagellum (arrow 3) in sperm (**2C**). Immunofluorescence staining of CaMKKβ (**2D**, green) was conducted; nuclei were stained with DAPI (**2E**, blue). Merged images of fluorescence with DAPI staining are shown in Fig **2F** (white arrows indicate regions containing CaMKKβ immunoreactivity: arrow 1: acrosome; arrow 3: principle piece; circle: midpiece). Scale bar: 10μm.

The presence of CaMKKs (α and β) was assessed by western-blotting and indirect immunofluorescence using specific primary antibodies against CaMKKα and CaMKKβ respectively. Proteins of the predicted size of CaMKKα (∼50kDa) (Fig 1A) and CaMKKβ (∼65kDa) (Fig 2A) were detected by western-blotting in chicken sperm as well as in positive controls (chicken skeletal muscle). CaMKKα was localized by immunofluorescence in the acrosome region and the midpiece, and at a much lower fluorescence in the principal piece of the flagellum (Fig 1F) whereas CaMKKβ was mainly localized in the principal piece of the flagellum and the acrosome region, and at a much lower fluorescence in the midpiece (Fig 2F).

**Fig 3:**
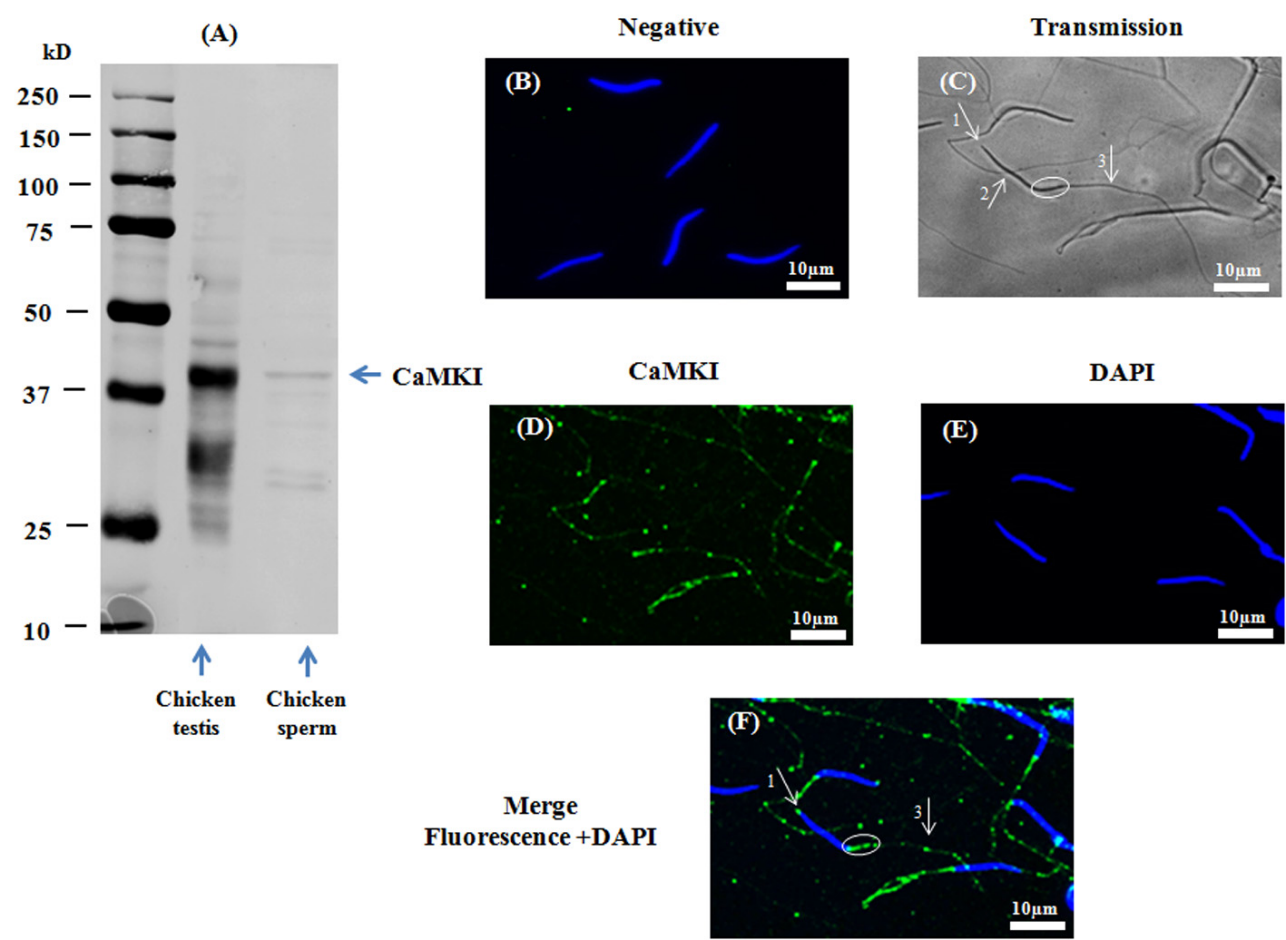
Presence and localization of CaMKI in chicken sperm. Chicken sperm lysates (45μg of protein) were analyzed by western blotting using anti- CaMKI as primary antibody. Cell lysates from chicken muscle (40μg of protein) were used as positive control. A band of approximately 41kDa for CaMKI was detected (**3A**). Indirect immunofluorescence of chicken sperm was carried out with the same antibody. Negative control: primary antibody was not added (**3B**). White arrows and circles in transmission images indicate areas of the acrosome (arrow 1), the nuclei (arrow 2), the midpiece (circles), and the principal piece of the flagellum (arrow 3) in sperm (**3C**). Immunofluorescence staining of CaMKI (**3D**, green) was conducted; nuclei were stained with DAPI (**3E**, blue). Merged images of fluorescence with DAPI staining are shown in Fig **3F** (white arrows indicate regions containing CaMKI immunoreactivity: arrow 1: acrosome; arrow 3: principle piece; circle: midpiece). Scale bar: 10μm.

A band of approximately 41kDa was detected using the anti-CaMKI antibody, showing the presence of CaMKI in chicken sperm (Fig 3A). CaMKI was detected by immunofluorescence in the acrosome region and the midpiece, as well as in the principal piece of the flagellum (Fig 3F).

**Fig 4:**
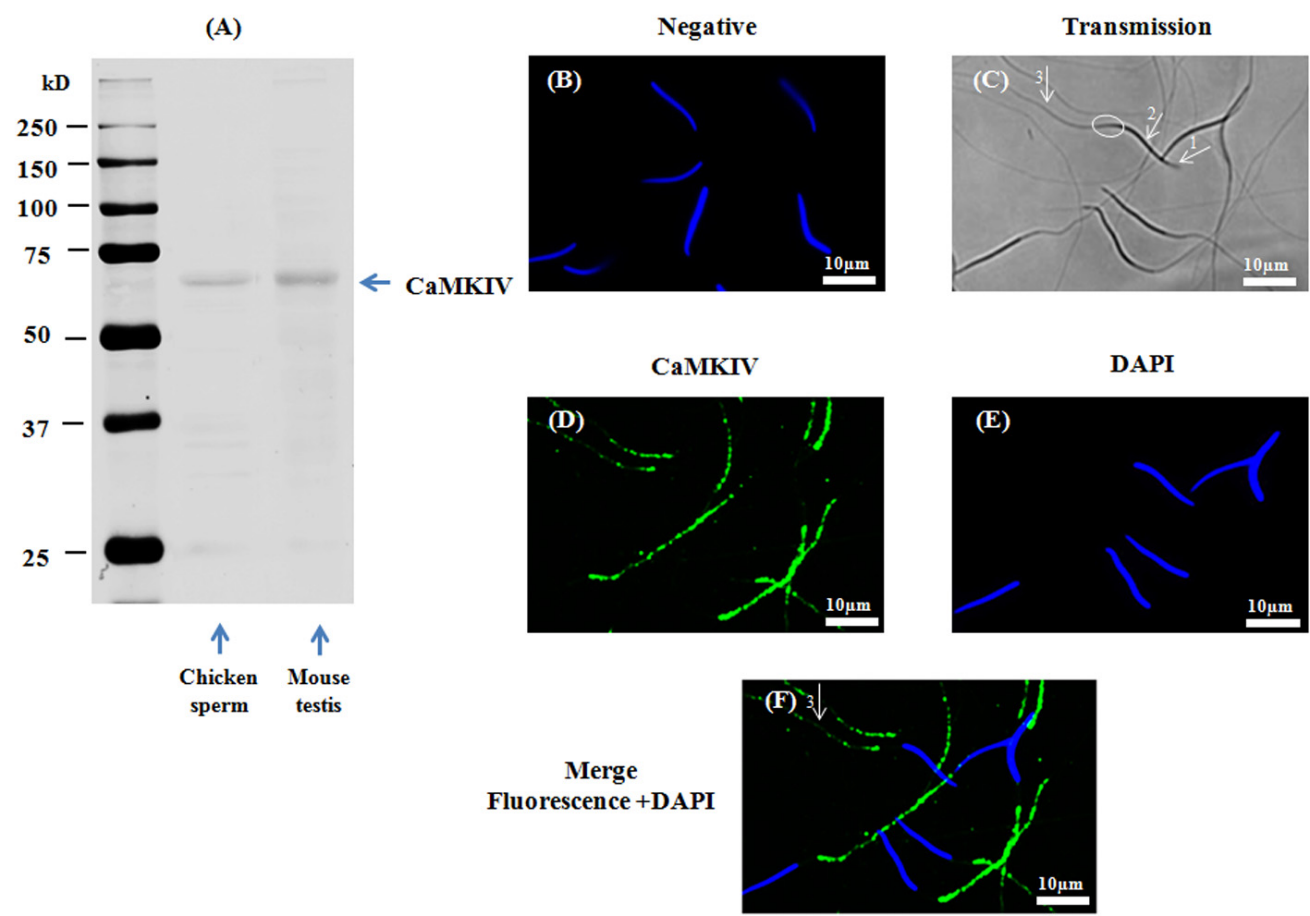
Presence and localization of CaMKIV in chicken sperm. Chicken sperm lysates (45μg of protein) were analyzed by western blotting using anti- CaMKIV as primary antibody. Cell lysates from mouse testis (40μg of protein) are used as positive control. A band of approximately 60kDa for CaMKIV was detected (**4A**). Indirect immunofluorescence of chicken sperm was carried out with the same antibody. Negative control: primary antibody was not added (**4B**). White arrows and circles in transmission images indicate areas of the acrosome (arrow 1), the nuclei (arrow 2), the midpiece (circles), and the principal piece of the flagellum (arrow 3) in sperm (**4C**). Immunofluorescence staining of CaMKIV (**4D**, green) was conducted; nuclei were stained with DAPI (**4E**, blue). Merged images of fluorescence with DAPI staining are shown in Fig **4F** (white arrows indicate regions containing CaMKIV immunoreactivity: arrow 3: principle piece). Scale bar: 10μm.

A band of approximately 60kDa was detected using the anti-CaMKIV antibody, showing the presence of CaMKIV in chicken sperm (Fig 4A). CaMKIV was detected by immunofluorescence only in the principal piece of the flagellum (Fig 4F).

Collectively, the results in Figs 1, 2, 3 and 4 demonstrate that CaMKKα and CaMKK β and their substrate CaMKI are present in different compartments of chicken sperm while CaMKIV is found in a unique compartment.

### Effect of extracellular Ca^2+^ on chicken sperm functions (motility, viability and AR) and AMPK phosphorylation, and CaMKI phosphorylation—Role of CaMKKs

#### a. Sperm functions (motility, viability and AR)

**Fig 5:**
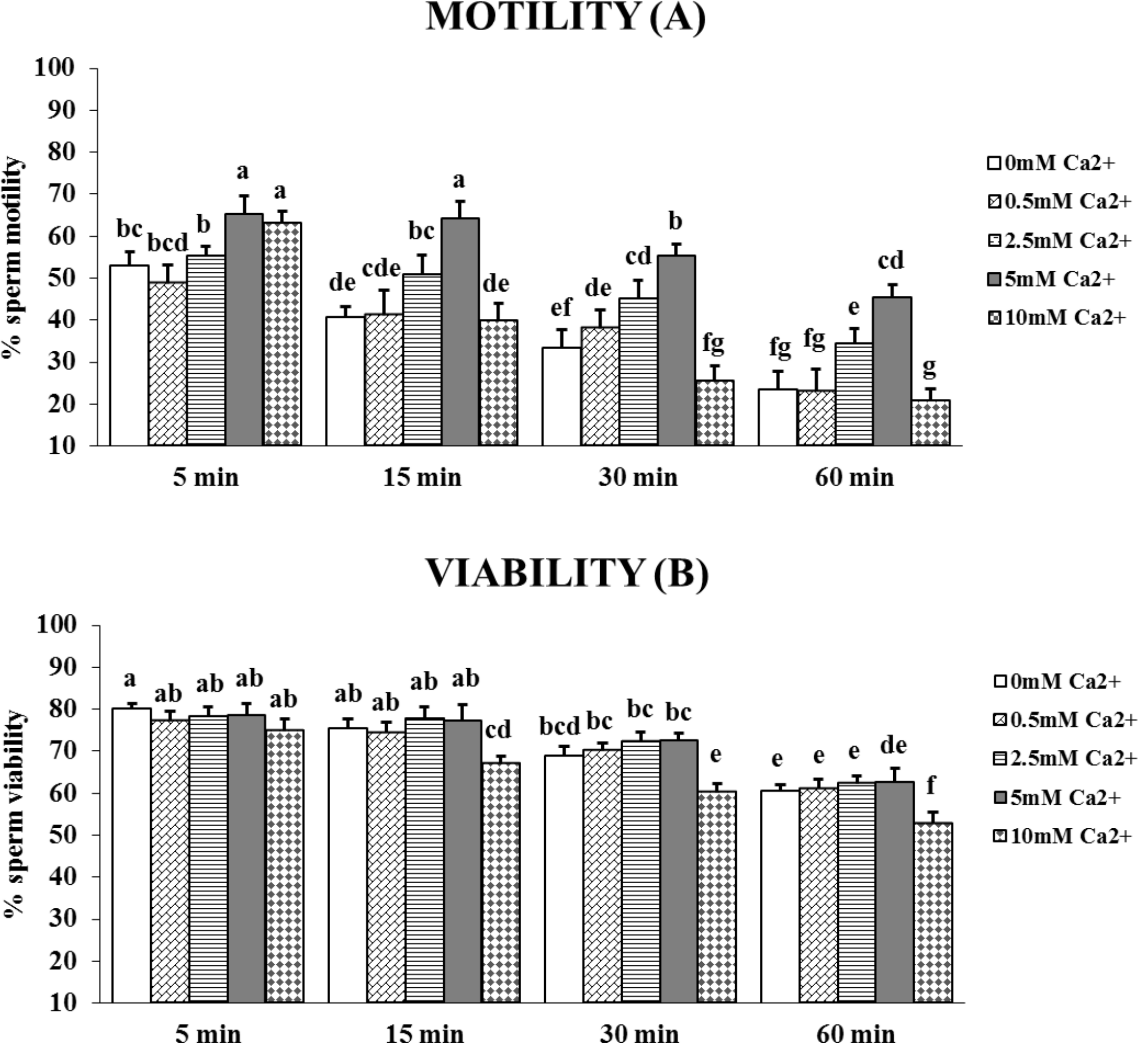
Effect of extracellular Ca^2+^ on chicken sperm motility and viability. Sperm were incubated for up to 60 min at 35°C in medium without Ca^2+^ (white) or containing different concentrations of Ca^2+^: 0.5mM (diagonal brick bars), 2.5mM (horizontal bars), 5mM (grey bar), 10mM (diamond bars). The percentages of motile **(A)** and viable **(B)** sperm after 5, 15, 30 and 60 min of incubation were determined as described in the Materials and Methods section. The results are expressed as mean ± SEM, n = 6. Different superscripts indicate statistically significant differences (P < 0.05).

The percentages of motile and viable cells were determined in sperm incubated for up to 1 hour in media containing increasing concentrations of extracellular Ca^2+^ (0.5, 2.5, 5 and 10mM). The maximum percentage of motile sperm was observed with 5mM Ca^2+^ (Fig 5), whatever the incubation time (mean increase from 23% to 92%, from 5 to 60 min). With 10mM Ca^2+^, after a rapid increase (of 19%) at 5 min, the percentage of motile sperm then decreased (Fig 5A). The percentage of viable sperm was not affected by Ca^2+^ concentrations up to 5mM, but was significantly decreased by 10mM Ca^2+^ after 15 and 30 min compared to the control without Ca^2+^ (Fig 5B). A significant decrease of viability was always observed after 1 hour of incubation. The 5mM Ca^2+^ concentration was the most efficient to preserve viability, and thus was retained for the further experiments.

**Fig 6:**
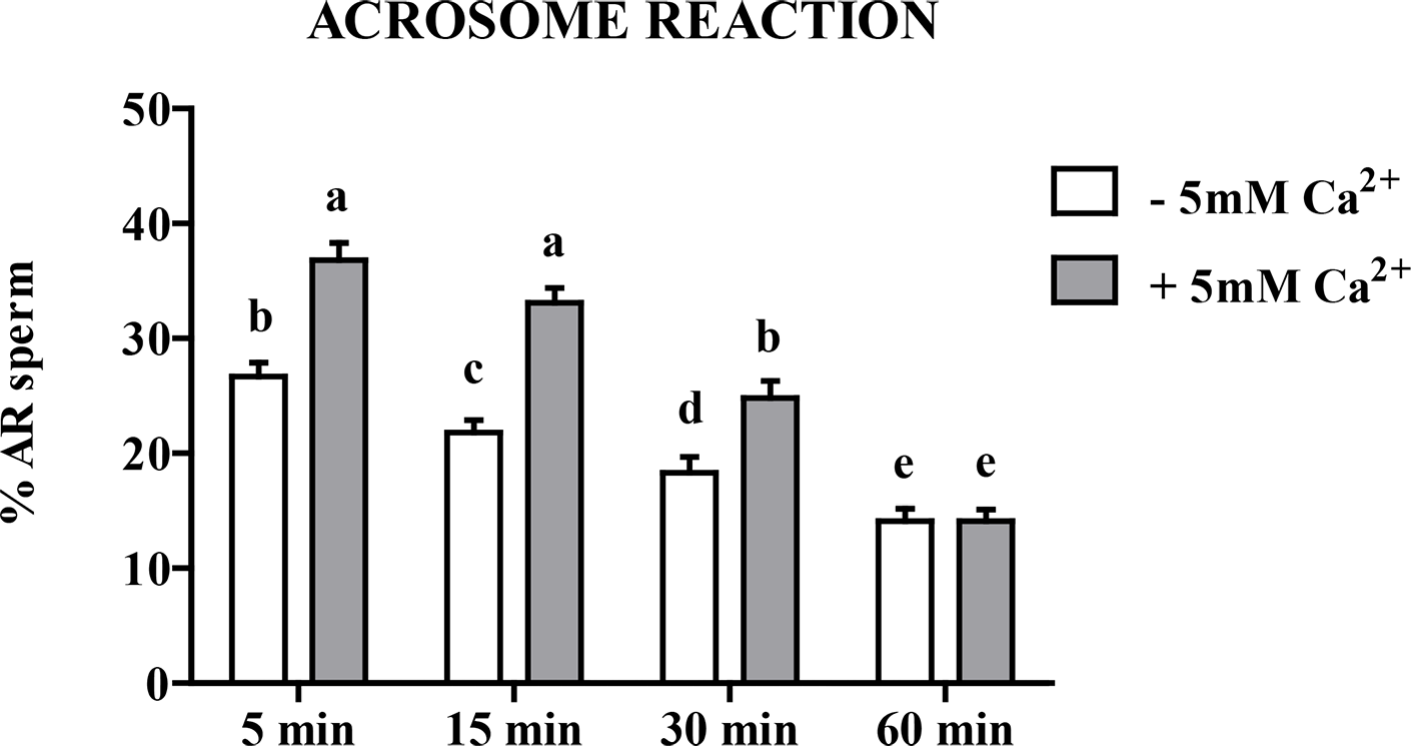
Effect of extracellular Ca^2+^ on chicken sperm AR. Sperm were incubated for up to 60 min at 35°C in medium not supplemented with Ca^2+^ (-5mM Ca^2+^, white bar) or containing 5mM Ca^2+^ (+5mM Ca^2+^, grey bar). The percentages of successful sperm AR after 5, 15, 30 and 60 min of incubation were determined as described in the Materials and Methods section. The results are expressed as mean ± SEM, n = 6. Different superscripts letters indicate statistically significant differences (P < 0.05).

Addition of 5mM Ca^2+^ increased the AR percentages by a difference of 38% at 5 min, 52% at 15 min, and 35% at 30 min over those values seen without added Ca^2+^ (Fig 6). As for viability and motility, monotonous significant decline in the percentage of AR was observed throughout the 60 min incubation and after 60 min the beneficial effect of Ca^2+^ was not visible anymore.

#### b. Role of CaMKKs in Ca^2+^-dependent AMPK phosphorylation in sperm

To assess the role of Ca^2+^ and CaMKKs in the regulation of AMPK, sperm were incubated with or without extracellular Ca^2+^ and in the presence or absence of the CaMKK inhibitor STO-609.

**Fig 7:**
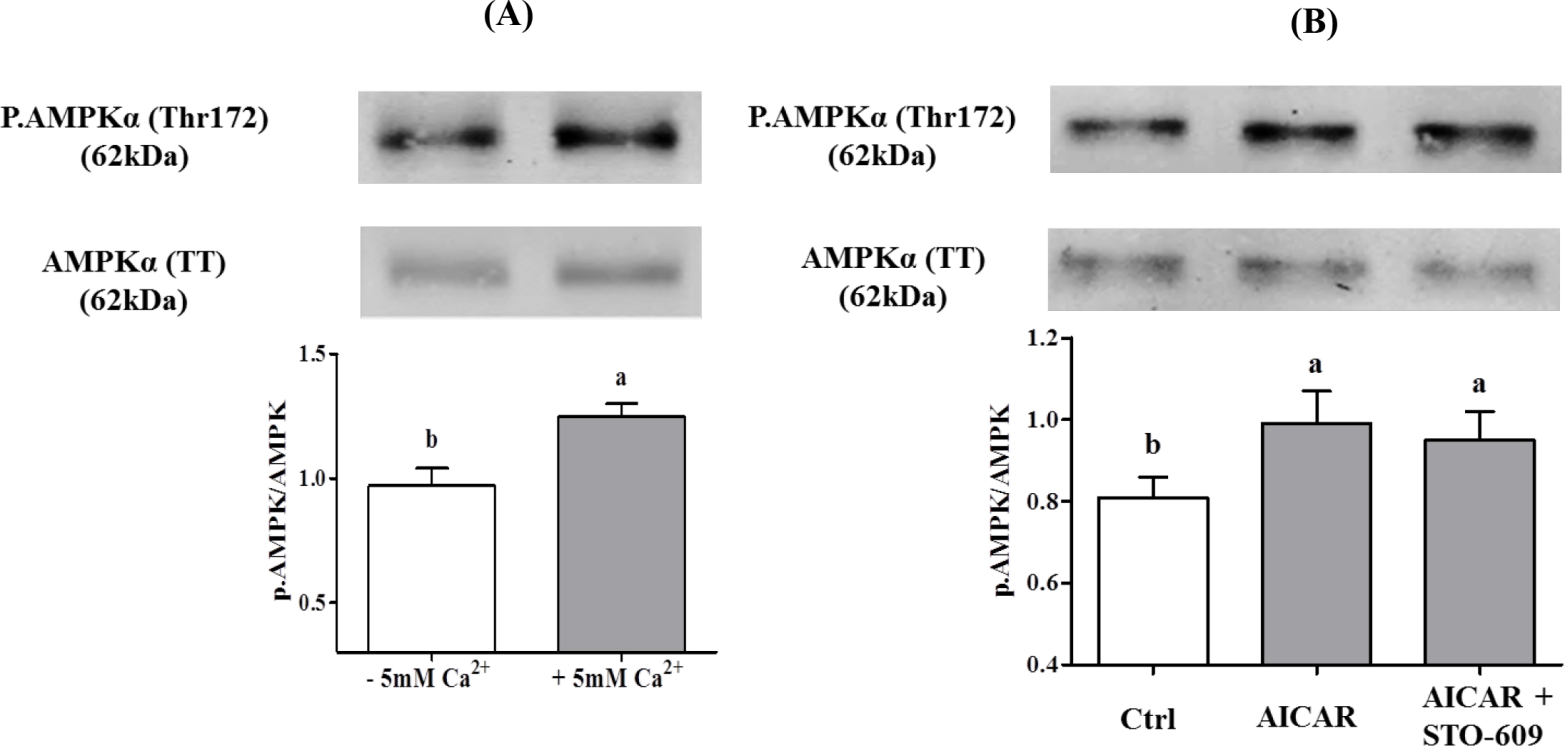
Effect of extracellular Ca^2+^ on the AMPK phosphorylation in chicken sperm. Sperm were incubated at 35°C in the –5mM Ca^2+^ or +5mM Ca^2+^ medium. Proteins from sperm lysates were analyzed by western-blotting using anti-phospho-Thr172-AMPKα or anti-AMPKα as primary antibody. Bands for phospho-Thr172-AMPKα were detected at 62kDa (top lanes). Total AMPKα (62kDa) was used as loading control (bottom lanes) and the phosphorylated protein AMPKα (Thr172)/total AMPKα ratio is shown at the bottom. **A)** Effects of increased intracellular 5mM Ca^2+^ on the AMPK phosphorylation after 5 min of incubation. **B)** Effect of STO-609 on AICAR induced AMPK phosphorylation: sperm were incubated with STO-609 for 5 min before adding 1mM AICAR and incubated 5 min more. The control: sperm incubated in the medium with DMSO. Results express the mean ± SEM of the mean from 6 different experiments. Different superscripts indicate statistically significant differences (P < 0.05).

In the absence of STO-609, the phosphorylation of AMPK was increased by 35% in response to extracellular Ca^2+^ presence (Fig 7A).

In order to rule out a possible direct action of STO-609 on AMPK, we tested whether 10μM of the inhibitor could inhibit the stimulatory action of AICAR on AMPK phosphorylation. The data (Fig 7B) show that this was not the case.

**Fig 8:**
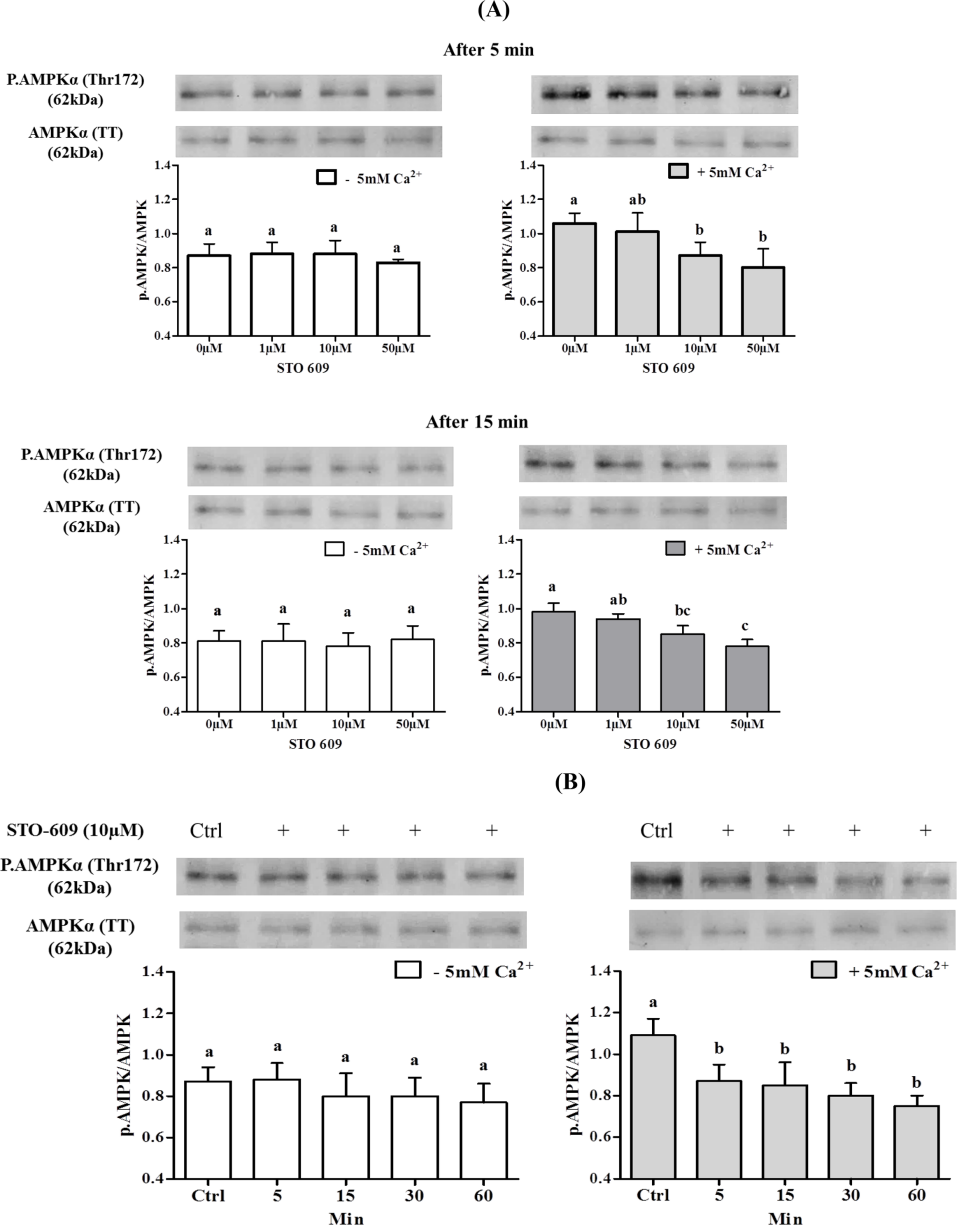
Effects of CaMKKs (STO-609) inhibitor on the AMPK phosphorylation in chicken sperm. Sperm were incubated at 35°C in the –5mM Ca^2+^ or +5mM Ca^2+^ medium with or without CaMKKs inhibitor STO-609 (1; 10; 50 μM) for 5 min and 15 min. Proteins from sperm lysates were analyzed by western-blotting using anti-phospho-Thr172-AMPKα or anti-AMPKα as primary antibody. Bands for phospho-Thr172-AMPKα were detected at 62kDa (top lanes). Total AMPKα (62kDa) was used as loading control (bottom lanes) and the phosphorylated protein AMPKα (Thr172)/total AMPKα ratio is shown at the bottom. **A)** Effects of different concentrations of STO-609 on the AMPK phosphorylation in sperm incubated for 5 min or 15 min. The control (Ctrl): sperm incubated with or without Ca^2+^ in the absence of STO-609. **B)** Effects of 10μM STO-609 on the AMPK phosphorylation in sperm incubated for 5, 15, 30, 60 min. The control (Ctrl): sperm incubated 0 min with or without Ca^2+^ in the absence of STO-609. Results express the mean ± SEM of the mean from 6 different experiments. Different superscripts indicate statistically significant differences (P < 0.05).

The involvement of Ca^2+^/CaMKKs on AMPK phosphorylation was analyzed next with increasing doses of the CaMKK inhibitor STO-609, with or without 5mM extracellular Ca^2+^. STO-609 inhibited in a dose-dependent fashion AMPK phosphorylation only in the presence of 5mM Ca^2+^ (Fig 8A). Moreover, the kinetics of STO-609 inhibition of AMPK phosphorylation in the presence of 5mM Ca^2+^ was very rapid (5 min) and lasted for over 60 min (Fig 8B).

#### c. Phosphorylation of CaMKI and CaMKIV in chicken sperm

**Fig 9:**
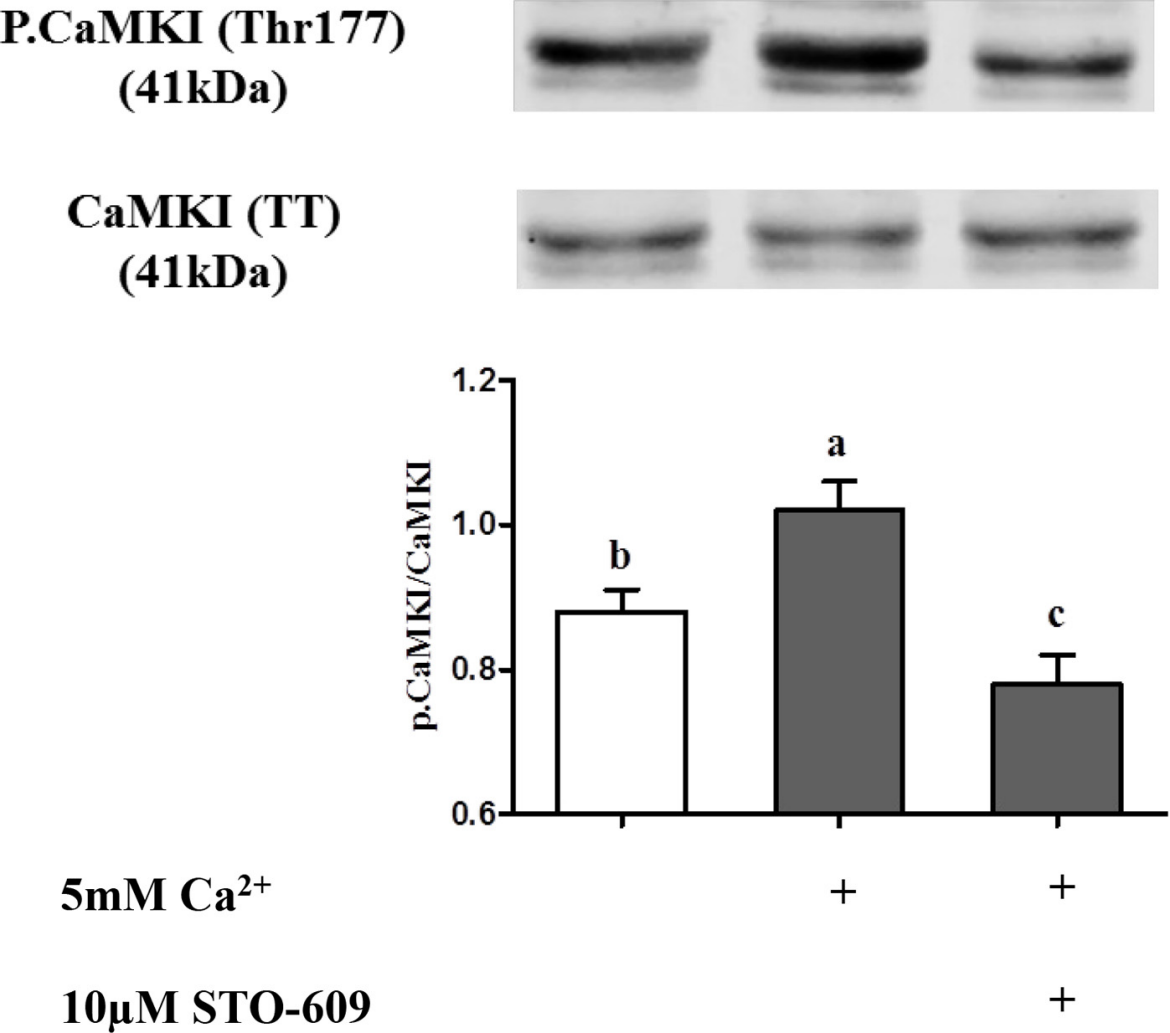
Effect of extracellular Ca^2+^ on the CaMKI phosphorylation in chicken sperm. Sperm incubated at 35°C for 5 min in the –5mM Ca^2+^ or +5mM Ca^2+^ medium with or without CaMKKs inhibitor STO-609. The control (Ctrl): sperm incubated in the absence both +5mM Ca^2+^ and 10μM STO-609. Proteins from sperm lysates were analyzed by western-blotting using anti-phospho-Thr177-CaMKI or anti-CaMKI as the primary antibody. Bands for phospho-Thr177-CaMKI were detected at 41kDa (top lanes). Total CaMKI (41kDa) was used as loading control (bottom lanes) and the phosphorylated protein CaMKI (Thr177)/total CaMKI ratio is shown at the bottom. Results express the mean ± SEM of the mean from 4 different experiments. Different superscripts indicate statistically significant differences (P < 0.05).

To further characterize Ca^2+^ signaling in sperm, we then analyzed the Ca^2+^-dependent CaMKI phosphorylation. As shown in Fig 9, CaMKI phosphorylation was increased by 23% in the presence of Ca^2+^ and this effect was reversed by pre-treatment with STO-609 which showed an additional decrease in CaMKI phosphorylation at a level significantly lower that the initial level without extracellular calcium (P˂0.05).

**Fig 10:**
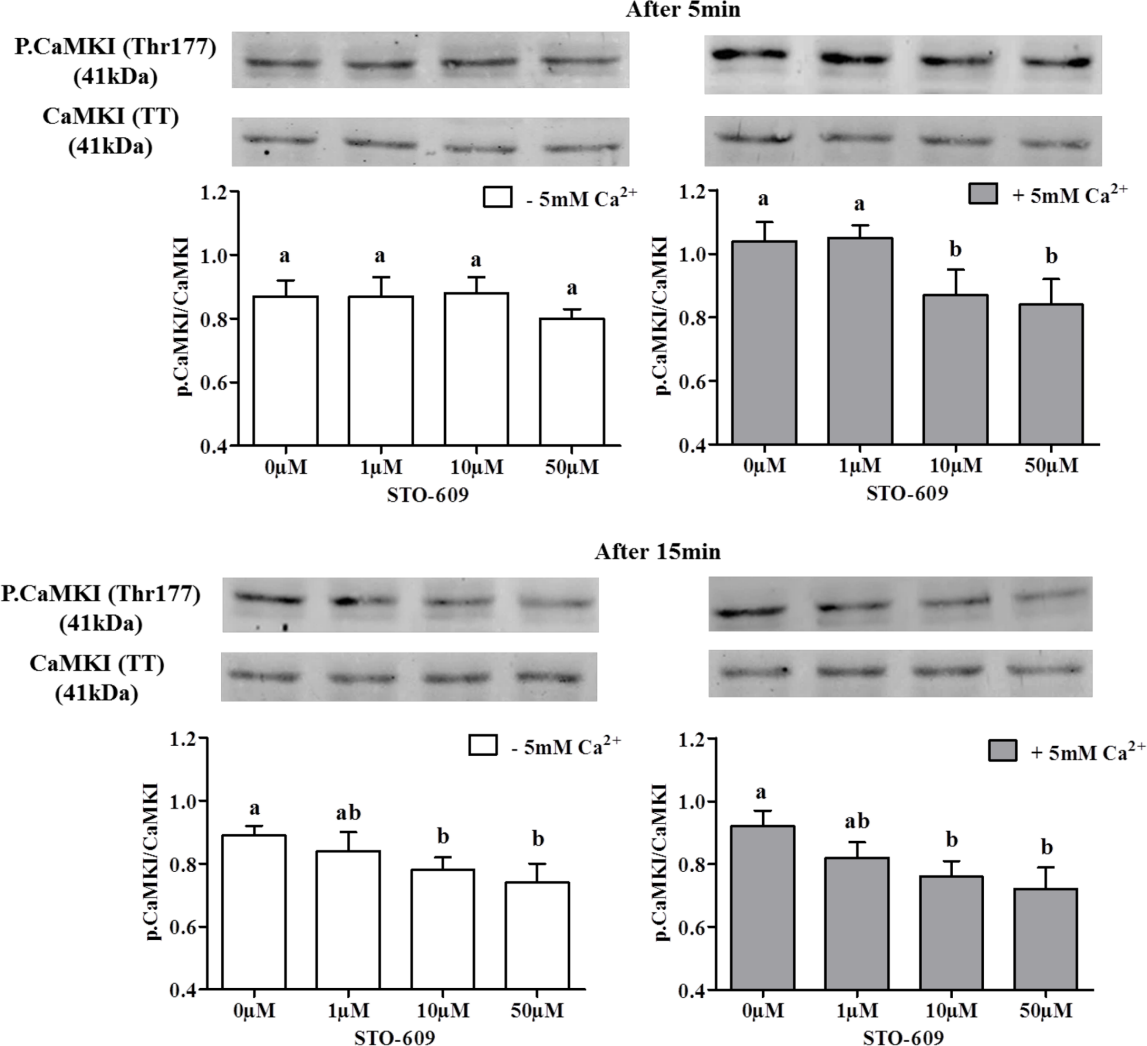
Effects of STO-609 on the CaMKI phosphorylation in chicken sperm. Sperm incubated at 35°C in the –5mM Ca^2+^ or +5mM Ca^2+^ medium with or without CaMKKs inhibitor STO-609 (0; 1; 10; 50 μM) at 5 min and 15 min. The control (Ctrl): sperm incubated with or without Ca^2+^ in the absence of STO-609. Proteins from sperm lysates were analyzed by western-blotting using anti-phospho-Thr177-CaMKI or anti-CaMKI as the primary antibody. Bands for phospho-Thr177-CaMKI were detected at 41kDa (top lanes). Total CaMKI (41kDa) was used as loading control (bottom lanes) and the phosphorylated protein CaMKI (Thr177)/total CaMKI ratio is shown at the bottom. Results express the mean ± SEM of the mean from 4 different experiments. Different superscripts indicate statistically significant differences (P<0.05).

**Fig 11:**
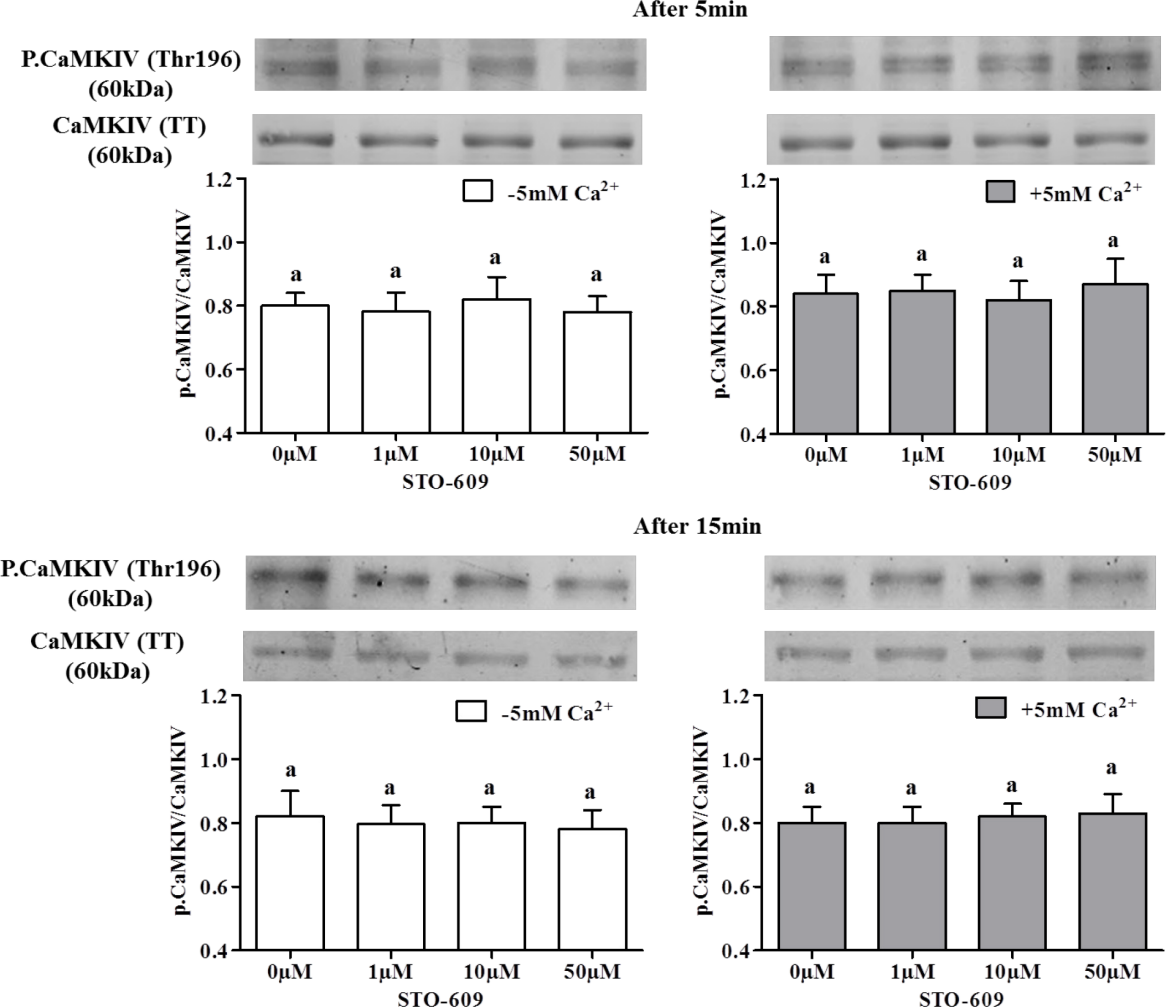
Effects of STO-609 on the CaMKIV phosphorylation in chicken sperm. Sperm incubated at 35°C in the –5mM Ca^2+^ or +5mM Ca^2+^ medium with or without CaMKKs inhibitor STO-609 (0; 1; 10; 50 μM) at 5 min and 15 min. The control (Ctrl): sperm incubated with or without Ca^2+^ in the absence of STO-609. Proteins from sperm lysates were analyzed by western-blotting using anti-phospho-Thr196-CaMKIV or anti-CaMKIV as the primary antibody. Bands for phospho-Thr196-CaMKIV were detected at 60kDa (top lanes). Total CaMKIV (60kDa) was used as loading control (bottom lanes) and the phosphorylated protein CaMKIV (Thr196)/total CaMKIV ratio is shown at the bottom. Results express the mean ± SEM of the mean from 4 different experiments. There were no significant differences between treatments (P<0.05).

In order to test the involvement of CaMKI and CaMKIV downstream of CaMKKs in the Ca^2+^-dependent AMPK phosphorylation, we studied CaMKI and CaMKIV phosphorylation at Thr177 and Thr196 respectively in the presence or absence of 5mM Ca^2+^ and/or of the CaMKK inhibitor 10μM STO-609. Results reported in Fig 10 show a dose-dependent effect of STO-609 on CaMKI phosphorylation after only 5 min of incubation in the presence of extracellular Ca^2+^, showing that CaMKKs inhibition decreases CaMKI phosphorylation. After 15 min, STO-609 inhibited CaMKI phosphorylation in the presence as well as in the absence of extracellular Ca^2+^, suggesting a secondary involvement of Ca^2+^ intracellular store in CaMKK catalysis of CaMKI phosphorylation. However, STO-609 did not significantly affect the phosphorylation of CaMKIV (Fig 11).

### Effects of the CaMKKs inhibitor STO-609 on sperm quality

To test whether AMPK phosphorylation by CaMKKs affects sperm biological parameters, sperm were incubated for 60 min with or without Ca^2+^ in the presence of various concentrations of the CaMKK inhibitor STO-609 (1μM, 10μM and 50μM). Over the whole 1-50μM range, STO-609 decreased the percentage of sperm motility and AR in a dose-dependent manner, both in the presence and absence of Ca^2+^. The initial motility and AR levels were even higher in the presence of Ca^2+^ (Tables 1–3). The highest STO-609 dose 50μM totally eliminated the acrosome reaction after 30 min of incubation (P< 0.05) (Table 3) and decreased the viable sperm by 17% after 15 min of incubation whatever Ca^2+^ was present in the medium or not (Table 4). Conversely, the use of 1μM and 10μM STO-609 did not affect the percentage of viable sperm (Table 4). Thus, the most informative STO-609 concentration for studying CaMKKs role on sperm quality (viability, motility parameters, and acrosome reaction) was 10μM which was chosen for the following studies.

**Table 1:** Effect of STO-609 treatment on the percentage of sperm motility (%).

Time of incubation	- 5mM Ca^2+^				+ 5mM Ca^2+^			
	STO-609 concentration (μM)							
	0 μM	1 μM	10 μM	50 μM	0 μM	1 μM	10 μM	50 μM
5 min	54.7±3.0^bc^	49.7±3.6^c^	39.2±4.7^d^	31.7±5.7^d^	63.7±4.2^a^	58.0±2.7^ab^	53.7±2.3^bc^	40.0±3.4^d^
15 min	40.5±4.7^cd^	30.7±5.3^de^	22.7±5.3^ef^	17.8±4.6^f^	64.5±4.8^a^	50.5±2.5^b^	41.5±2.1^c^	34.0±3.6^d^
30 min	31.8±6.1^bc^	23.5±6.4^c^	9.3±2.1^d^	8.3±2.1^d^	51.0±4.0^a^	37.3±3.2^b^	32.3±4.5^bc^	25.5±3.6^c^
60 min	21.7±5.5^bc^	13.5±4.9^cd^	8.2±2.8^de^	4.5±1.3^e^	37.8±4.9^a^	29.3±4.4^ab^	23.3±4.0^b^	18.0±3.4^c^

Values represent means ± SEM from 6 different experiments.

Different superscripts indicate statistically significant differences of values within a row (P<0.05).

**Table 2:** Effects of CaMKK inhibitor STO-609 (10μM) on sperm motility parameters.

Motility parameters	- 5mM Ca^2+^				+ 5mM Ca^2+^			
	5 MIN		60 MIN		5 MIN		60 MIN	
	Control	STO	Control	STO	Control	STO	Control	STO
Motile (%)	54.7±3.0^b^	39.2±4.7^c^	21.7±5.5^d^	8.2±2.8^e^	63.7±4.2^a^	53.7±2.3^b^	37.8±4.9^c^	23.3±4.0^d^
Progressive (%)	19.3±2.4^b^	13.7±1.9^c^	5.8±1.9^d^	1.7±1.0^e^	28.0±3.6^a^	21.5±2.4^b^	12.0±2.8^c^	5.7±1.0^d^
Rapid(%)	35.5±3.9^ab^	25.0±4.7^c^	11.3±2.7^d^	3.8±1.4^e^	45.3±4.9^a^	33.5±2.4^b^	23.7±3.9^c^	12.8±2.2^d^
VAP (μm/s)	58.3±4.3^b^	57.0±4.5^b^	56.8±4.0^b^	43.3±4.9^c^	70.0±5.7^a^	68.1±4.8^a^	58.6±3.4^b^	46.5±4.3^c^
VSL (μm/s)	43.5±4.8^bc^	42.0±4.9^bc^	43.7±4.1^bc^	32.6±3.2^d^	56.9±6.5^a^	55.0±5.1^a^	45.5±3.7^b^	35.6±3.8^cd^
VCL (μm/s)	108.6±5.9^a^	109.0±6.4^a^	105.1±5.5^a^	92.7±4.2^b^	117.5±6.1^a^	115.1±4.8^a^	112.5±3.4^a^	107.2±5.3^a^
STR (%)	70.8±2.7^bc^	70.7±2.9^bc^	64.2±2.7^d^	60.0±2.3^d^	77.3±1.7^a^	77.3±1.8^a^	73.2±1.6^b^	69.5±0.6^c^
LIN (%)	39.7±2.7^bc^	38.5±2.9^bc^	38.7±2.1^bc^	32.2±2.3^d^	47.3±3.2^a^	46.8±3.1^a^	40.5±2.5^b^	34.8±2.0^cd^

Values represent means ± SEM from 6 different experiments.

Different superscripts indicate statistically significant differences of values within a row (P<0.05).

**Table 3:** Effect of STO-609 treatment on chicken sperm AR (%).

Time of incubation	- 5mM Ca^2+^				+ 5mM Ca^2+^			
	STO-609 concentration (μM)							
	0 μM	1 μM	10 μM	50 μM	0 μM	1 γM	10 μM	50 μM
5 min	26.6±1.5^b^	22.3±1.5^cd^	20.4±1.0^d^	13.8±1.1^f^	31.0±1.5^a^	28.2±1.5^ab^	26.4±1.7^b^	17.1±1.0^e^
15 min	20.0±1.5^b^	16.4±0.8^c^	12.2±1.3^d^	8.4±1.0^e^	32.2±1.1^a^	21.5±1.5^b^	16.9±1.3^c^	10.5±0.5^de^
30 min	17.3±1.9^b^	12.7±0.5^c^	9.2±0.8^d^	6.4±0.6^e^	24.3±1.8^a^	15.3±1.3^b^	9.1±0.9^d^	5.4±0.7^e^
60 min	12.9±1.5^a^	9.2±0.9^b^	5.3±0.8^c^	4.2±0.5^d^	14.0±0.7^a^	8.4±0.7^b^	5.6±0.5^c^	4.0±0.9^d^

Values represent means ± SEM from 6 different experiments.

Different superscripts indicate statistically significant differences of values within a row (P<0.05).

**Table 4:** Effect of STO-609 treatment on chicken sperm viability (%).

Time of incubation	- 5mM Ca^2+^				+ 5mM Ca^2+^			
	STO-609 concentration (μM)							
	0 μM	1 μM	10 μM	50 μM	0 μM	1 μM	10 μM	50 μM
5 min	79.3±1.7^a^	79.3±1.2^a^	78.8±1.6^a^	76.3±2.3^a^	80.3±2.1^a^	79.4±0.8^a^	79.2±1.6^a^	76.6±2.2^a^
15 min	77.6±2.4^a^	72.2±1.9^a^	70.2±1.0^a^	64.6±2.2^b^	78.9±1.4^a^	68.8±1.9^a^	69.3±1.8^a^	63.5±2.1^b^
30 min	70.4±1.4^a^	69.6±2.1^a^	64.1±2.3^b^	63.3±1.5^b^	72.5±1.2^a^	69.4±1.1^a^	62.1±1.2^b^	62.5±1.7^b^
60 min	64.6±1.7^a^	64.6±1.7^a^	58.8±1.4^b^	54.9±1.5^cd^	62.9±1.8^a^	64.3±1.6^a^	57.2±1.4^bc^	54±1.1^d^

Values represent means ± SEM from 6 different experiments.

Different superscripts indicate statistically significant differences of values within a row (P<0.05).

#### a. Sperm motility

10μM STO-609 reduced most sperm motility parameters (% motile, % rapid cells, μm/s VAP, μm/s VSL, μm/s VCL, % STR, and % LIN) in both the presence or absence of Ca^2+^ (Table 2).

In the presence or absence of Ca^2+^, 10μM STO-609 significantly decreased motile, rapid and progressive cells after only 5 min of incubation compared to the control, while the other parameters, VAP, VSL, VCL, LIN and STR, were significantly decreased compared to the control after 60 min of incubation. There was about 28% reduction of the percentage of motile sperm in the medium without 5mM Ca^2+^ at 5 min and 62% at 60 min, and 16% reduction of the percentage of motile sperm in the medium with 5mM Ca^2+^ at 5 min and 38% at 60 min. Moreover, the motility parameters were even higher in the presence than in the absence of Ca^2+^.

#### b. Sperm acrosome reaction

The addition of 10μM STO-609 significantly decreased the sperm acrosome reaction capacity in a time-dependent manner, and acrosome reaction was dramatically reduced after 60 min of incubation in the presence or absence of calcium in the medium (Table 3). In the absence of calcium, STO-609 promoted a significant decrease of the acrosome reaction rate of ~ 23% and ~ 59% after 5 min and 60 min respectively, and in the presence of 5mM Ca^2+^, the respective decreases after 5 min and 60 min were ~15% and ~60% (Table 3). Thus the STO-609 effects were similar in the presence or absence of Ca^2+^, but the initial level of AR was higher in its presence (P<0.05).

#### c. Sperm viability

After 5 min of incubation in 10μM STO-609, the percentage of sperm viability assessed with SYBR14/PI was not affected. However, after 60 min of incubation in 10μM STO-609 significantly decreased sperm viability by 9% in both the absence and presence of calcium in the medium compared to their respective controls. After 60 min of incubation, the rate of viable cells decreased by 20% for the controls and by 26% with 10μM STO-609 in both media compared to the measurements taken after 5 min (Table 4). The 10μM STO-609 effect was again the same whether Ca^2+^ was present or not.

## Discussion

Calcium plays fundamental and diversified roles in cell metabolism. The sperm are highly motile cells with a silent genome [3–4] but they are very reactive in engaging in motility, exocytosis, and fertilization. Using chicken sperm as a model, the present study describes the involvement of actors of the calcium-dependent signaling pathways involved in AMPK regulation and activation of sperm functions.

For the first time in germ cells, we localized CaMKKα and CaMKKβ and showed that CaMKKβ and phospho-AMPK [6] were localized in the same subcellular compartments: mainly in the acrosome and flagellum. The CaMKK downstream substrate CaMKI was also identified in germ cells and showed the same subcellular distribution as the other CaMKK form, CaMKKα: mainly in the acrosome and midpiece. Another potential CaMKK substrate, CaMKIV, was also found but only in the flagellum. The presence of extracellular Ca^2+^ in the sperm medium increased by 29% AMPK phosphorylation and 22% CaMKI phosphorylation and sperm functions as expected in case of activation of the Ca^2+^/CaMKKs/CaMKI pathway while the inhibition of this pathway through STO-609 returned to the basal levels observed in absence of Ca^2+^. AMPK is a central actor in energy homeostasis regulation having a key role in activation of sperm function [6]. Activation of AMPK can be induced by phosphorylation through the action of CaMKKs. Here we identified CaMKKα and CaMKKβ in chicken sperm using western-blotting and we further localized them by immunocytochemistry. CaMKKs are serine threonine kinases that are activated when bound to Ca^2+^/CaM complexes and are thus members of the CaM kinase family [15]. CaMKKs (α and β) are upstream of AMPK, especially CaMKKβ that can directly phosphorylate AMPK-Thr172 [1–2]. The localization in the same subcellular compartments of CaMKKβ and phospho-AMPK supports the view that CaMKKβ directly phosphorylates AMPK in chicken sperm, as previously shown in other cell types [1–2]. On the other hand, CaMKKα exhibited a quite different subcellular localization (in the acrosome and midpiece), very similar to that of CaMKI. These findings suggest different roles for CaMKKα and β, each of them acting mainly with different preferential substrates: CaMKKβ > AMPK and CaMKKα > CaMKI (>AMPK).

Experiments with the presence or absence of extracellular Ca^2+^ and/or of the selective CaMKK inhibitor STO-609 provided further evidence that Ca^2+^/ CaMKK signaling is effective in the stimulation of AMPK and CaMKI phosphorylations. Both CaMKI and AMPK phosphorylations were stimulated in the presence of extracellular Ca^2+^ concentrations corresponding to normal physiological levels in chicken oviducts [49]. The AMPK activation by CaMKKs in the presence of extracellular Ca^2+^ was close to that which had been previously observed with boar sperm [19]. In the absence of extracellular Ca^2+^, the phospho-Thr172-AMPK was not affected by STO-609 even at the highest concentration of 50μM, showing the dependence of CaMKKs towards extracellular Ca^2+^ for AMPK phosphorylation. STO-609 is a selective inhibitor of both CaMKKα and CaMKK β [50], but it could inhibit other kinases such as AMPK itself when used at high concentrations *in vitro* [1–2, 18]. Such action was ruled out here since AICAR-induced phosphorylation of AMPK was not affected by STO-609. Altogether, we conclude that extracellular Ca^2+^-induced AMPK activation in chicken sperm is mediated through CaMKKs.

CaMKK-dependent AMPK activation was found to be very rapid in chicken sperm, in less than 5 min of incubation at 35°C. This is consistent with our previous results showing fast AMPK activation by AICAR in the absence of extracellular Ca^2+^ [6]. These actions are much faster than in boar sperm and might be related to the fact that chicken sperm are very rapid for all signaling pathways studied up to now, possibly due to lack of the so called “capacitation” process observed in many mammals [19]. Unlike mammalian sperm, chicken sperm acquire their motility and part of their fertilization ability as soon as they leave the testis [51–52]. Birds’ sperm reside for a very long time in the female genital tract (up to 3 weeks for chicken) before acrosome reaction and fertilization occur [53]. They do not show capacitation or sperm hyperactivation before the acrosome reaction that occurs in less than 5 min in vitro [32]. The signaling pathways involved in acrosome reaction and in motility of chicken sperm are also activated *in vitro* in less than 5 min [5–6]. We thus suggested that these highly differentiated cells with an “inactive genome” could be a model to study “non-genomic” rapid signaling on different metabolic functions.

Furthermore, we show that CaMKI phosphorylation was increased upon extracellular Ca^2+^ stimulation, and that this effect was inhibited when the cells were pre-treated with STO-609. This strongly suggests that CaMKKs catalyzed CaMKI phosphorylation. Interestingly, the STO 609-dependent decrease of CaMKI phosphorylation was observed even in the absence of extracellular calcium, albeit more slowly (15 min instead of 5 min). This suggests that CaMKI can be activated in chicken sperm in response to the release of internal stores of cellular Ca^2+^. The sensitivity of CaMKKβ to STO-609 being 5-fold higher than that of the CaMKKα [54], it might be that the slower CaMKI phosphorylation in the absence of extracellular calcium is mediated through CaMKKα activation by Ca^2+^ internal stores, whereas the faster CaMKI phosphorylation in the presence of extracellular Ca^2+^ is mediated through CaMKKβ activation. However, we did not observe any change of CaMKIV phosphorylation while using STO-609. This suggests that the localization of CaMKIV in the sperm flagellum is consistent with a putative role in chicken sperm motility regulation, but not through the mediation of CaMKKs.

We also demonstrate here that appropriate doses (10μM) of the CaMKK inhibitor, STO-609, decreased chicken sperm motility and acrosome reaction without affecting sperm viability. STO-609 rapidly decreased sperm AR as well as several sperm motility parameters (% motile, % rapid, % progressive) after 5 min of incubation in the presence of extracellular Ca^2+^, in parallel with a decrease in AMPK and CaMKI phosphorylations. This suggests that CaMKKs affect chicken sperm motility and acrosome reaction through AMPK and CaMKI activation. STO-609 also quickly (after 5 min) decreased chicken sperm functions in the absence of extracellular Ca^2+^. However, in the absence of extracellular Ca^2+^, the decrease of CaMKI phosphorylation was only observed after 15 min of incubation, indicating the involvement of intracellular Ca^2+^ in CaMKI phosphorylation through CaMKKs, but the decrease was slower than in presence of extracellular Ca^2+^. It can thus be hypothesized that the direct Ca^2+^/CaMKKs/AMPK and indirect Ca^2+^/CaM/CaMKKs/CaMKI/AMPK signaling pathways are rapidly involved together in chicken sperm functions in the presence of extracellular Ca^2+^. In its absence, the Ca^2+/^CaM/CaMKKs/CaMKI signaling pathway could be involved in chicken sperm motility, and AR in another way independent of extracellular Ca^2+^ entry after 15 minutes of incubation. Alternatively, the levels obtained without extracellular Ca^2+^ could also take other routes that do not pass through Ca^2+^/CaMK/CaMKKs/CaMKI signaling pathway since the decrease of sperm functions observed from 5 to 15 min of incubation in the presence of STO-609 reached a basal level lower in the absence of Ca^2+^ than in Ca^2+^ presence. Both CaMKI and AMPK can be phosphorylated by CaMKKs, suggesting direct and/or indirect AMPK activation through its Thr172 phosphorylation. However, the possibility that the Ca^2+^/CaM complex directly affects sperm functions cannot be ruled out since the addition of W-7 or trifluoperazine (inhibitors of CaM) inhibited the motility of fowl spermatozoa [42]. Moreover, CaM is present in both the principal piece and the acrosome of mouse sperm [39] and is involved in various Ca^2+^ dependent sperm functions in other species [55–57], but through still undefined pathways. Ca^2+^ is also known as a regulator of the predominant flagellar adenylate cyclase variant, soluble adenylyl cyclase (sAC) [58–60], but independently of CaM in mammals [61].

**Fig 12:**
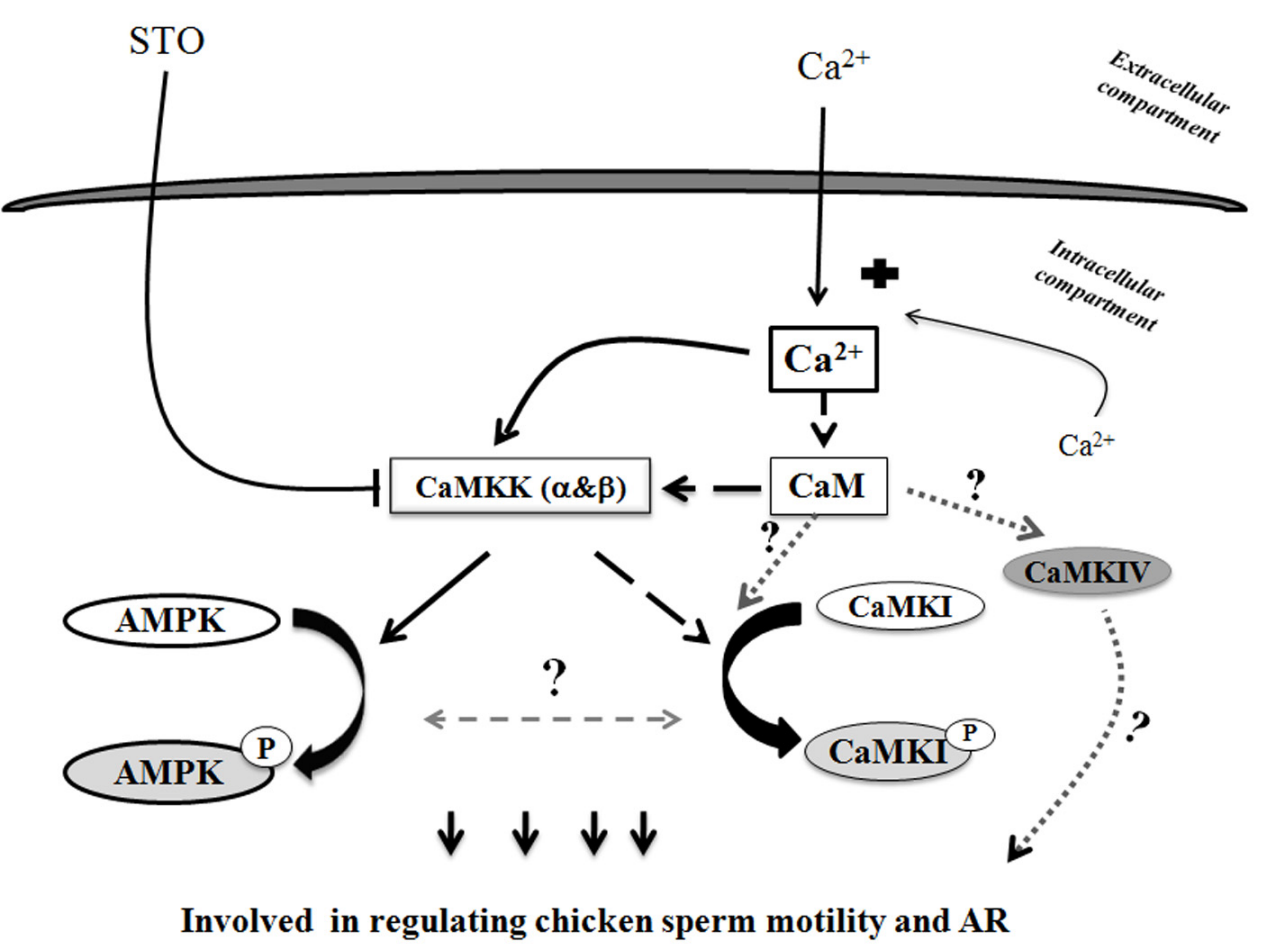
Proposed scheme of Ca^2+^ signaling pathways leading to regulation of AMPK activity in avian sperm. This schematic diagram shows potential mechanisms of calcium role through CaMKK stimulation in chicken sperm. They all activate AMPK and CaMKI, which leads to metabolic improvements, leading themselves to the control of sperm functions. Solid arrows and blocked arrows illustrate more established relationships between stimuli, signals, improved metabolic status and control of avian sperm functions. Solid black arrows indicate Ca^2+^/CaMKKs/AMPK signaling pathway. Dashed black arrows indicate Ca^2+^/CaM/CaMKKs/CaMKI signaling pathway. Curved arrows (black) indicate AMPK phosphorylation, CaMKI phosphorylation; dashed grey arrows with a question mark are used for hypotheses.

All our results suggest the action of new actors in the regulation of sperm functions that are activated in the presence of extracellular calcium and are summarized in Fig 12. The increase of intracellular Ca^2+^ levels from extracellular Ca^2+^ entry in the sperm activates the Ca^2+^/CaMKKs/AMPK signaling pathway with further stimulating effects on sperm motility and AR. In the absence of extracellular calcium, the Ca^2+^/CaMKK signaling pathway is less solicited to phosphorylate AMPK and other ways of action are opened. In all cases, the signaling pathways are quicker with extracellular calcium AMPK pathway (5 min) than with stimulation by intracellular calcium CaMKI pathway (15 min). Taken together, our data bring new information that highlight the complex relationships between calcium signaling pathways and energetic metabolism needed to ensure germ cell functions.

### Conclusion

This study provides the first evidence of the localization and action of CaMKK α and β and CaMKI on extracellular calcium dependent AMPK regulation in sperm cells. We have also shown a new signaling pathway, Ca^2+^/CaM/CaMKKs/CaMKI, involved in birds’ sperm functions which can be activated with extracellular calcium. These results improve our knowledge of signal transduction in highly reactive cells with silent genomes.
